# An upper bound for the background rate of human extinction

**DOI:** 10.1038/s41598-019-47540-7

**Published:** 2019-07-30

**Authors:** Andrew E. Snyder-Beattie, Toby Ord, Michael B. Bonsall

**Affiliations:** 1 University of Oxford, Mathematical Ecology Research Group, Department of Zoology, Oxford, OX1 3SZ UK; 2 University of Oxford, Future of Humanity Institute, Faculty of Philosophy, Oxford, OX1 1PT UK

**Keywords:** Natural hazards, Planetary science

## Abstract

We evaluate the total probability of human extinction from naturally occurring processes. Such processes include risks that are well characterized such as asteroid impacts and supervolcanic eruptions, as well as risks that remain unknown. Using only the information that *Homo sapiens* has existed at least 200,000 years, we conclude that the probability that humanity goes extinct from natural causes in any given year is almost guaranteed to be less than one in 14,000, and likely to be less than one in 87,000. Using the longer track record of survival for our entire genus *Homo* produces even tighter bounds, with an annual probability of natural extinction likely below one in 870,000. These bounds are unlikely to be affected by possible survivorship bias in the data, and are consistent with mammalian extinction rates, typical hominin species lifespans, the frequency of well-characterized risks, and the frequency of mass extinctions. No similar guarantee can be made for risks that our ancestors did not face, such as anthropogenic climate change or nuclear/biological warfare.

## Introduction

Out of all species that have existed, over 99% are now extinct^1^. Although human activity is dramatically increasing extinction rates for many species^2^, species extinctions were regular occurrences long before humanity emerged. Many of these extinctions were caused by gradual environmental shifts, evolutionary arms races, or local interspecific competition^3,4^, while others were abrupt, being part of global mass extinctions caused by asteroid impacts, volcanism, or causes as of yet to be identified^5,6^. Could such a catastrophe befall our own species? If so, are the risks greater from natural or anthropogenic sources?

Here, we evaluate the natural ‘background’ extinction rate for *Homo sapiens*. This means considerations of anthropogenic risks such as climate change and nuclear weapons are excluded from our estimates, although these clearly pose existential threats to our own species as well as others. Indeed, it has been hypothesized that the great majority of human extinction risk comes from anthropogenic sources^7,8^. But by limiting our analysis to natural risks that our predecessors also faced, we can draw on data spanning many thousands (or millions) of years. Obtaining bounds on natural extinction rates also enables an indirect and partial test of the hypothesis that anthropogenic risks are greater than natural ones, as sufficiently low natural extinction risk will imply higher relative risks from anthropogenic sources.

Estimating such an extinction rate directly is impossible. We have no examples of *Homo sapiens* extinction, so the most directly relevant data are non-existent. An alternative approach would be to enumerate the different types of naturally occurring hazards (e.g. asteroids, supervolcanoes), estimate their independent probability of causing extinction, and then use these probabilities to derive an aggregate extinction rate. However, this method has its own shortcomings. Beyond the great uncertainties around the probabilities of each risk, there could also be unknown risks that fail to be included. It would be hard to say with confidence that any list of risks had captured all natural hazards to humanity.

We can bypass these problems by instead considering the length of time that humanity has survived so far^9,10^. This survival time can be used to estimate an upper bound on the extinction rate from all natural sources combined, including from sources for which we remain unaware. However, this approach could be subject to a particular form of sample bias known as an observation selection bias. These observer selection biases occur when a sample is not representative of all outcomes, but rather a subset of outcomes that are compatible with the existence of the observers^11^. For example, if human existence required a 10 million year (Myr) period of evolution free from asteroid impacts, any human observers will necessarily find in their evolutionary history a period of 10 Myr that is free of asteroid impacts, regardless of the true impact rate. Inferring a rate based on those 10 Myr could therefore be misleading, and methods must to be used to correct for this bias^12^.

Using data from archeological and fossil records, we place an upper bound on the natural rate of human extinction. We then test this model against possible forms of observer selection bias, and demonstrate that the data are unlikely to be severely biased due to these effects. We finally cross-check our conclusions against alternative forms of data, including mammalian extinction rates, the temporal ranges of other hominin species, and the frequency of potential catastrophes and mass extinctions.

## Bounding the Extinction Rate Based on Age of Humanity

Anatomically modern human fossils in Ethiopia have been dated to 195 ± 5 thousand years ago (kya)^13^. A more recent fossil discovery in Morocco of an anatomically modern human has been dated to 315 ± 34 kya^14,15^ (though the fossil may exhibit more primitive neurocranial and endocranial morphology). Given that *Homo sapiens* has existed for hundreds of thousands of years, what can we infer about our background rate of extinction?

Assuming that we share a common extinction rate with our predecessors, we can rule out rates that are too high to be compatible with this track record of survival. As our aim is to construct an upper bound, we can set aside the possibility that modern human technology, habitat range, and population size have reduced a number of natural extinction risks. The upper bound is only violated if we have reason to believe current extinction rates are higher than those our predecessors faced. Since we exclude anthropogenic risks from our analysis, we also set aside the majority of the ways in which this could be the case, although we acknowledge there exist boundary cases between purely natural and anthropogenic risks (e.g. a naturally emerging disease could be spread further by modern technology). Ultimately the scope of the upper bound is limited to all risks that have remained constant (or have been reduced) over the past few hundred thousand years.

### Likelihood of extinction rates

Analysis of taxonomic survivorship curves and temporal ranges for a wide variety of taxa suggest that extinction probabilities can be approximated well by assuming a constant risk of extinction over time^16–18^. Under this model, extinction can be represented by the exponential distribution with constant extinction rate *μ*. The probability that humanity goes extinct before time *t* is given by the cumulative distribution function *P*(*T* ≤ *t*) = 1 − *e*^−*μt*^, where *T* is the random variable denoting the longevity of our species. Conversely, the probability that humanity makes it beyond time *t* is *P*(*T* ≥ *t*) = *e*^−*μt*^.

**Figure 1:**
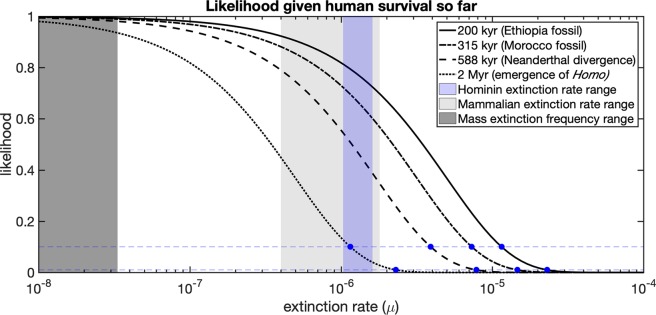
Likelihood of extinction rates given our track record of survival so far, with estimated ranges of Hominin extinction rates, mammalian extinction rates, and mass extinction frequency included for reference. Blue horizontal lines indicate likelihood of 10% and 1%. Rates exceeding 6.9 × 10^−5^ are ruled out even with the most conservative data. Extending humanity’s track record of survival to match older fossils, the divergence with *Homo neanderthalensis*, or the origin of *Homo* creates even stricter bounds.

We want to evaluate the likelihood of an extinction rate *μ*, given the observation that humanity has lasted up to time *t* (so we know that the total longevity of humanity *T* ≥ *t*). This can be evaluated as the likelihood function ${\mathcal L} (\mu |T\ge t)={e}^{-\mu t}$. We compute the likelihood of extinction rates between 10^−8^ and 10^−4^ given a number of different plausible starting dates for *Homo sapiens* outlined in Fig. 1 and Table 1.

**Table 1:** Survival times and resulting upper bounds.

Track Record (*t*)	Starting Point	Value of *μ* with relative likelihood of…		
		10^−1^	10^−2^	10^−6^
200 kyr	Anatomically modern humans^13^	1.2 × 10^−5^	2.3 × 10^−5^	**6.9 × 10**^**−5**^
315 kyr	Anatomically modern humans^14,15^	7.3 × 10^−6^	1.5 × 10^−5^	4.4 × 10^−5^
588 kyr	Neanderthal divergence^19,20^	3.9 × 10^−6^	7.8 × 10^−6^	2.4 × 10^−5^
2 Myr	Emergence of *Homo*^21,22^	**1.2 × 10**^**−6**^	2.3 × 10^−6^	6.9 × 10^−6^

Assuming a 200 thousand year (kyr) survival time, we can be exceptionally confident that rates do not exceed 6.9 × 10^−5^. This corresponds to an annual extinction probability below roughly 1 in 14,000. The relative likelihood for such high extinction rates are below 10^−6^ (one in a million) when compared to a rate of 10^−8^. If we assume that our track record extends further, this upper bound becomes stronger. Using the fossil dated to 315 ka as a starting point for humanity gives an upper bound of *μ* < 4.4 × 10^−5^, corresponding to an annual extinction probability below 1 in 22,800. Using the emergence of *Homo* as our starting point pushes the initial bound back a full order of magnitude, resulting in an annual extinction probability below 1 in 140,000.

We can also relax the one in million relative likelihood constraint and derive less conservative upper bounds. An alternative bound would be rates with relative likelihood below 10^−1^ (1 in 10) when compared to the baseline rate of 10^−8^. If we assume humanity has lasted 200 kyr, we obtain a bound of *μ* < 1.2 × 10^−5^, corresponding to an annual extinction probability below 1 in 87,000. Using the 2 Myr origin of *Homo* strengthens the bound by an order of magnitude in a similar way and produces annual extinction probabilities below 1 in 870,000.

It is worth noting that this model can be generalised to allow for a varying extinction rate over time *μ*(*t*), so that the probability of surviving past time *t* is given by *P*(*T* ≥ *t*) = *e*^−Θ(*t*)*t*^, where ${\rm{\Theta }}(t)=(1/t){\int }_{0}^{t}\,\mu (s)ds$. The upper bound on Θ(*t*), the average extinction rate over the interval, can then be calculated in the same way as for the constant rate model.

## Observation Selection Effects

The data on humanity’s survival time could be subject to survivorship bias. If early *Homo sapiens* requires a long period of time to develop the intellectual machinery needed to make scientific observations, then such observations could not include short evolutionary histories, regardless of the extinction rate. The amount of information we could derive from a long track record of survival would therefore be limited due to this observation selection effect. Such a track record could indicate a low extinction rate, or be the byproduct of lucky ancestors surviving high extinction rates long enough to beget progeny capable of making scientific observations. One might therefore object that the bounds on the extinction rate we have estimated are too low^12,23^. Here, we examine and respond to this concern.

### Models to quantify potential sample bias

To model observation selection bias, let us assume that after *Homo sapiens* first arises another step must be reached. This could represent the origin of language, writing, science, or any relevant factor that would transition early humans into the reference class of those capable of making observations (we call this step ‘observerhood’). Let this step be a random variable denoted *S*, with cumulative distribution function *F*_*S*_(*t*). As we are examining natural risks, we assume that *S* and *T* are independent. The probability that humanity survives long enough to reach observerhood status (via intelligence, language, writing, science, etc) can be found with the following integral:

1 $$P(T > S)={\int }_{0}^{\infty }\,{f}_{T}(t){F}_{S}(t)dt$$

where *f*_*T*_(*t*) = *μe*^−*μt*^, the probability of extinction at time *t*. We evaluate an adjusted likelihood function ${ {\mathcal L} }^{\ast }(\mu |T > t)$, denoting that we are taking the likelihood of an extinction rate *μ* given that humanity has survived to time *t*, and the fact that we are conditioning on the existence of observers such that *T* > *S*. This results in the adjusted likelihood function:

2 $${ {\mathcal L} }^{\ast }(\mu |T > t)=P(T > t|T > S,\mu )$$

3 $$=\,\frac{1}{c}{\int }_{t}^{\infty }\,{f}_{T}(s){F}_{S}(s)ds$$

where *c* = *P*(*T* > *S*) is a normalising constant. We evaluate a model with four variations for the observerhood step: a model in which observerhood occurs as a single event that has a constant rate over time, a model with an increasing rate over time, a model with multiple steps, and a model where observerhood simply requires a fixed amount of time.

If desired, we could more crisply define this observerhood property as the ability for a species to collect reliable data on its own track record of survival (e.g. via fossil dating) and analyse it. When correcting for observation selection effects, we are simply conditioning on the fact that our species has developed the ability to conduct this analysis. The observerhood property need not invoke consciousness or be the property of a biological species—a machine estimating a parameter would need to account for observer selection bias if its ability to make such estimates were correlated with the parameter in question.

### Model 1: Single step, constant rate

Our first model assumes that observerhood has a constant rate of occurrence *θ*, so that *S* is exponentially distributed with cumulative distribution function: *F*_*S*_(*t*) = 1 − *e*^−*θt*^. This model describes a process in which the transition from early humans into observers occurs by chance as a single step. This could represent the hypothesis that hierarchical language emerged in humans as the byproduct of a chance mutation^24^. With this model, the probability that observers arrive before extinction is *P*(*T* > *S*) = *θ*(*θ* + *μ*)^−1^. Our likelihood function can be analytically derived:

4 $${ {\mathcal L} }^{\ast }(\mu |T > t)=(\frac{\theta +\mu }{\theta }){\int }_{t}^{\infty }\,\mu {e}^{-\mu s}(1-{e}^{-\theta s})ds$$

5 $$=\,(\frac{\theta +\mu }{\theta }){e}^{-\mu t}-(\frac{\mu }{\theta }){e}^{-(\mu +\theta )t}$$

### Model 2: single step, increasing rate

Our second model similarly assumes that a single step is needed but that the rate of observerhood increases over time. This model could represent increasing population size or population density, which could in turn drive cultural evolution and increase the probability of such a step^25^. We represent this with a Weibull distribution with cumulative distribution function ${F}_{S}(t)=1-{e}^{-{(\theta t)}^{k}}$ where *k* > 1 indicates increasing rate over time (when *k* = 1, this is the same as the exponential in Model 1). We use numerical integration to evaluate the likelihood function.

### Model 3: multiple steps, constant rate

Our third model assumes that there are multiple steps that need to occur in a sequence in order to get observers. This could represent more incremental development of tools, culture, or language. We assume that each step is exponentially distributed with rate *θ*, so that the timing of the final *k*th step follows an Erlang distribution with cumulative distribution function:

6 $${F}_{S}(t)=1-\sum _{n=0}^{k-1}\,\frac{1}{n!}{e}^{-\theta t}{(\theta t)}^{n}.$$

Note that when *k* = 1, the distribution is the same as the exponential in Model 1. We use numerical integration to evaluate the likelihood function.

### Model 4: fixed time requirement

Our final model assumes that it takes a fixed amount of time *τ* to reach observerhood. This is an extreme model that allows for no chance, but could represent a gradual and deterministic accumulation of traits. The probability that observerhood has been reached before time *t* is therefore *F*_*S*_(*t*) = 1_[*t*>*τ*]_, the characteristic function that takes the value 1 when *t* > *τ* and 0 otherwise. The probability that humanity survives past time *τ* is 1 − *F*_*T*_(*τ*) = *e*^−*μτ*^. Our likelihood function of *μ* is:

7 $${ {\mathcal L} }^{\ast }(\mu |T > t)=\frac{1}{{e}^{-\mu \tau }}{\int }_{t}^{\infty }\,\mu {e}^{-\mu s}{1}_{[s > \tau ]}ds$$

8 $$=\,{e}^{-\mu (t-\tau )}.$$

This likelihood expression can also be derived using the memoryless property of the exponential. It is worth noting that the fixed time model is a limiting case for both the increasing rate model and the multiple steps model. Taking the limit of Model 2 as *k* → ∞ results in a fixed time model with *τ* = *θ*^−1^. Similarly, Model 3 converges to a fixed time model as the number of steps increases and the expected time of each step decreases (having infinitely many steps in the limit, each of which is infinitely short).

### Results of sample bias models

**Figure 2:**
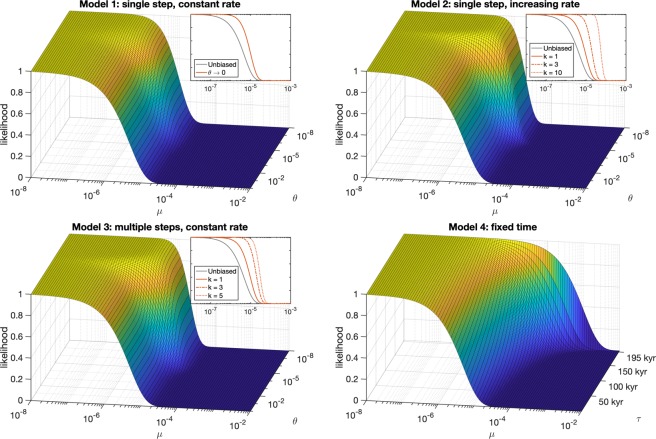
Models of observer selection bias. Surface plots show likelihood for combinations of *μ* and *θ* (where *k* = 3 for Models 2 and 3) or *τ* in Model 4. Upper righthand plots show how likelihood shifts when *θ* → 0 in Model 1, and for a variety of *k* values in Models 2 and 3. For the first three models, the unbiased model is recovered for large *θ*, and results start to become biased as the expected observerhood time approaches humanity’s track record of survival. However, even as *θ* → 0, the bias is limited, and the likelihood of rates exceeding 10^−4^ remains at zero. This is only violated in the final fixed time model, or in models 2 and 3 when *k* is sufficiently large.

We evaluate the likelihood of extinction rates between 10^−8^ and 10^−2^, given a human survival time of 200 kyr and a wide range of different rates at which observers could originate (Fig. 2). The first thing to note about the first three models is that when the observerhood rates are sufficiently rapid, the likelihood function converges to the unbiased version in the previous section. This can be verified by taking limits: for all of the models as *θ* → ∞ (or *τ* → 0 in the case of the fixed time model), ${ {\mathcal L} }^{\ast }(\mu |T > t)\to {e}^{-\mu t}$. If observerhood is expected to occur quickly, then we can take a 200 kyr track record of survival at face value and estimate the extinction rate without observation selection bias.

However, as the observerhood rates decrease to the point where the expected observerhood time approaches an order of magnitude close to 200 kyr, observer selection bias emerges. Rates that were previously ruled out by our track record of survival are assigned higher likelihoods, since a portion of the track record is a necessity for observers (Fig. 2). For example in Model 1, when *θ* = 2 × 10^−4^ (corresponding to an expected observerhood time of 20 kyr), the relative likelihood of *μ* = 6.9 × 10^−5^ is increased by a factor of 2.3 (from 10^−6^ to 2.3 × 10^−6^). To get a likelihood of 10^−6^ (corresponding to the most conservative upper bound), the rate must be set at 7.3 × 10^−5^ (see all edited bounds in Table 2). Interestingly though, this effect is limited. Even as observerhood rates slow to the point where expected observerhood time greatly exceeds 200 kyr (for example exceeding 20 billion years), the revised upper bounds remain within a factor of 2 of the original bounds. The stricter the bound, the weaker the potential bias: for example the 10^−6^ likelihood bound is only changed by a factor of about 1.2 in the limit as *θ* → 0. Although there would be some sample bias, there is a hard ceiling on how much our track record of survival can be distorted by observation selection effects.

**Table 2:** Upper bounds of *μ* with model 1 bias.

*θ*	${\boldsymbol{ {\mathcal L} }}{\boldsymbol{=}}{{\bf{10}}}^{{\boldsymbol{-}}{\bf{1}}}$	${\boldsymbol{ {\mathcal L} }}{\boldsymbol{=}}{{\bf{10}}}^{{\boldsymbol{-}}{\bf{2}}}$	${\boldsymbol{ {\mathcal L} }}{\boldsymbol{=}}{{\bf{10}}}^{{\boldsymbol{-}}{\bf{6}}}$
Unbiased	1.2 × 10^−5^	2.3 × 10^−5^	6.9 × 10^−5^
2 × 10^−4^	1.3 × 10^−5^	2.5 × 10^−5^	7.3 × 10^−5^
2 × 10^−5^	1.7 × 10^−5^	3.1 × 10^−5^	8.1 × 10^−5^
*θ* → 0	1.9 × 10^−5^	3.3 × 10^−5^	8.3 × 10^−5^

The reason slow rates of observerhood have a limited impact on our estimates is because if the extinction rate were exceptionally high, the lucky humans that do successfully survive to observerhood will have achieved such a status unusually quickly, and therefore will still observe a very short track record of survival. A long track record of survival is therefore still sufficient to rule out high extinction rates paired with low observerhood rates. We can demonstrate this by examining the typical time it takes for lucky survivors to reach observerhood, assuming a high extinction rate and a low observerhood rate. For example, in the single step constant rate model when *θ* = 10^−6^ (corresponding to an expected observerhood time of 1 Myr) and *μ* = 10^−3^ (corresponding to a typical extinction time of 1000 years), the expected observerhood time conditional on these high extinction rates is 1000 years. A typical observer will thus still have a very short track record of survival. Models with increasing rates or multiple steps exhibit the same property, although the bias is larger depending on parameter *k*. For both model 2 and 3 with *θ* = 10^−6^, *μ* = 10^−3^, and *k* = 2 (parameters normally corresponding to an expected observerhood time of 830 kyr for Model 2 and 2 Myr for model 3), the high extinction rates will still result in a typical observer emerging unusually early and having only about a 2000 year track record of survival. This can be also seen in Fig. 2 where for Models 1, 2, and 3, the likelihood of high extinction rates exceeding 10^−4^ are still assigned low likelihood regardless of *θ*.

However, severe observer selection bias can occur in Models 2 and 3 as *k* becomes larger, shaping the observerhood distribution such that early observerhood is vanishingly unlikely and late observerhood almost guaranteed. In the most extreme case this is represented by the fixed time model, where the probability of observerhood jumps from 0 to 1 when *t* = *τ* (the fixed time model is also the limiting case when *k* → ∞). If that fixed amount of time is long enough (say, exceeding 190 or 195 kyr), a 200 kyr track record of survival is no longer sufficient to rule out extinction rates greater than 10^−4^. This result occurs as the fixed time model prohibits any possibility of observerhood occurring unusually quickly. Any lineage of *Homo sapiens* lucky enough to survive long enough to obtain observer status must necessarily have a survival time greater than *τ*, which means that being an observer with a survival time of *τ* conveys zero information about the extinction rate.

For numerous reasons, we find the fixed time model to be implausible. Virtually all biological and cultural processes involve some degree of contingency, and there is no fundamental reason to think that gaining the ability to make scientific observations would be any different. To illustrate a comparison, let us consider a world in which the extinction rate is 10^−4^ (averaging one extinction every 10,000 years), but observerhood status takes a fixed 200 kyr. Under this model, humanity successfully surviving long enough to reach observer status is an event with 1 in 200 million chance. Given observation selection bias, we cannot rule out the possibility of rare events that are required for our observations. But we could ask why a 1 in 200 million chance event could not also include the possibility that modern human observers would emerge unusually rapidly. Language, writing, and modern science are perhaps highly unlikely to develop within ten thousand years of the first modern humans, but it seems exceptionally overconfident to put the odds at fewer than 1 in 200 million.

A similar line of reasoning can be applied to determine whether the increasing rate and multiple step models with high *k* are reasonable. We test this by asking what parameters would be needed to expect a 200 kyr track record of survival with an extinction rate at our conservative upper bound of *μ* = 6.9 × 10^−5^. For the increasing rate model, observerhood is expected after 203 kyr with *θ* = 10^−7^ and *k* = 14 and for the multiple step model, observerhood is expected after 190 kyr with *θ* = 10^−7^ and *k* = 16. Although these models do not assign strictly zero probability to early observerhood times, the probabilities are still vanishingly small. With an increasing rate and these parameters, observerhood has less than a one in a trillion chance of occurring within 10,000 years (3.4 × 10^−14^), and about 1% chance of occurring within 100,000 years. With multiple steps and these parameters, observerhood has less than one in a trillion chance of occurring within 10,000 years (5.6 × 10^−17^), and less than a 0.02% chance of occurring within 100,000 years. In a similar fashion to the fixed time model, we feel that these models exhibit unrealistic levels of confidence in late observerhood times.

Although the plausibility of the fixed time (or nearly fixed time) models is hard to test directly, the wide variance in the emergence of modern human behavior across geography offers one source of data that can test their plausibility. The Upper Palaeolithic transition occurred about 45 kya in Europe and Western Asia, marked by the widespread emergence of modern human behaviour^25^ (e.g. symbolic artwork, geometric blades, ornamentation). But strong evidence exists for the sporadic appearance of this modern human behaviour much earlier in parts of Africa^26,27^, including evidence of artwork and advanced tools as early as 164 kya^28^. Although numerous factors could have prevented the Upper Palaeolithic transition from occurring quickly, the fact that some human communities made this transition more than 100 kyr earlier than the rest of humanity indicates that a much earlier development trajectory is not entirely out of the question.

In summary, observer selection effects are unlikely to introduce major bias to our track record of survival as long as we allow for the possibility of early observers. Deceptively long track records of survival can occur if the probability of early observers is exceptionally low, but we find these models implausible. The wide variance in modern human behavior is one source of data that suggests our track record is unlikely to be severely biased. We can also turn to other sources of indirect data to test for observer selection bias.

## Testing the Bound with Indirect Data

We cross check our upper bound against four other sources of data: mammalian extinction rates, survival times of other human species, rates of potential catastrophes, and mass extinction rates. Although these alternative data do not directly predict the background extinction rate of *Homo sapiens* per se, the rates of extinction are likely generated by similar processes and thus enable an indirect test of the upper bound. If our upper bound is sound (not biased by observer selection effects or otherwise flawed), we can make testable predictions that it will be (A) broadly consistent with the extinction rates for similar species, and (B) not exceeded by the rate of potential catastrophes or mass extinctions. As the extinction rate of other species and catastrophes many millions of years ago have little bearing on our ability to make scientific observations, these data are also less subject to potential observer selection bias.

### Mammalian extinction rates

We first evaluate whether the upper bound is consistent with extinction rates for a typical mammalian species. Using fossil record data, median extinction rates for mammals have been estimated as high as 1.8 extinctions per million species years (E/MSY)^2^, or equivalently *μ* = 1.8 × 10^−6^. Other estimates using fossil record data range from 0.165 extinctions per million genus years^17^ to 0.4 E/MSY for Cenozoic mammals^18^. Alternative methods using molecular phylogeny suggest a much lower rate of 0.023 E/MSY for mammals^29^ and rates of 0.219–0.359 E/MSY for primates^30^, although these methods have been criticized^31^. All of these estimated background rates are consistent with our upper bound. It is worth noting that *Homo sapiens* may be at lower extinction risk than a typical mammalian species due to a large habitat range, large population size, and having a generalist diet, which are all traits that militate against extinction risk (whereas long generation times and large body mass are sometimes correlated with increased extinction risk)^32,33^.

### Hominin survival times

Next, we evaluate whether the upper bound is consistent with the broader hominin fossil record. There is strong evidence that *Homo erectus* lasted over 1.7 Myr and *Homo habilis* lasted 700 kyr^21^, indicating that our own species’ track record of survival exceeding 200 kyr is not unique within our genus. Fossil record data indicate that the median hominin temporal range is about 620 kyr, and after accounting for sample bias in the fossil record this estimate rises to 970 kyr^22^. Although it is notable that the hominin lineage seems to have a higher extinction rate than those typical of mammals, these values are still consistent with our upper bound. It is perhaps also notable that some hominin species were likely driven to extinction by our own lineage^34^, suggesting an early form of anthropogenic extinction risk.

### Individual sources of extinction risk

The upper bound can also be evaluated against the frequency of events that could pose extinction risks (examples provided in Table 3). If any particular risk (such as those from asteroid impacts) is known to have a higher rate than our bound of 6.9 × 10^−5^, this could undermine and potentially falsify our hypothesis. We evaluate the frequencies of four types of potential disasters for which credible quantitative estimates exist: asteroid impacts, supervolcanic eruptions, stellar explosions, and vacuum collapse. All of these risks have been estimated to occur with a frequency well below our bound (Table 3), with the exception of smaller supervolcanic eruptions. Recent work has suggested the frequency of eruptions ejecting >10^3^ km^3^ of material exceeds our upper bound of 6.9 × 10^−5^ with a recurrence time of 17 kyr^35^.

**Table 3:** Catastrophe frequency estimates.

Risk	Frequency
Asteroid ≥1 km	500 kyr^36^
Asteroid ≥5 km	6 Myr^37^
Supervolcano 10^3^ km^3^	1.1 Myr^38^
Supervolcano 10^3^ km^3^	17 kyr^35^
Flood Basalt	32 Myr^39^
Gamma Ray Burst	170 Myr^40^
Supernovae	100 Myr^41^
Vacuum collapse	>1 Gyr^42^

However, it is important to note that the smaller eruptions within this category do not necessarily have a high probability of causing human extinction. The most severe eruption of the past 2 million years occurred just 74 kya, and it is unclear whether the human population at the time was at risk of extinction. Some argue that the human population suffered a major bottleneck at the same time as the eruption^43^, although this theory remains controversial^44^. Some climate records averaged over decades fail to observe a severe volcanic winter in Africa at the time^45^ and archaeological evidence shows that human communities in South Africa thrived both before and after the eruption^46^ (although these data are not sufficient to rule out a severe short-lived catastrophe followed by a fast recovery in population). More conclusively, most members of the Hominidae family did not suffer population bottlenecks around the time, with the possible exception of Eastern chimpanzees and Sumatran orangutans^47^. The lack of dramatic evidence suggesting other species extinctions or bottlenecks undercuts the possibility that humanity’s survival was highly improbable and is observed only due to observation selection effects. However, a handful of substantially larger flood basalt events have taken place over the past 250 Myr that have been linked to mass extinctions^39,48^. These events occur with a frequency of roughly once every 20–30 Myr, much more infrequently than smaller eruptions. If we assume that human extinction is threatened only from larger volcanic eruptions well exceeding 10^3^ km^3^, then none of the risk frequencies we have catalogued come within an order of magnitude of the conservative upper bound.

Similarly, impacts from smaller asteroid around 1 km in diameter may not have a high probability of causing human extinction. Although it is hard to estimate the consequences of such impacts, some researchers have argued that such impacts would fall below the threshold for a global catastrophe^49^. Impacts that disperse enough dust and sulphites to significantly disrupt photosynthesis occur much more rarely, with an estimated frequency of about 15 Myr years^49^. If we assume human extinction is only threatened by these more severe impacts exceeding 5 km, each of these catastrophe frequencies falls well below even our most optimistic bound of 1 in 870,000 chance of extinction per year.

### Mass extinction frequency

A mass extinction is marked by substantially increased extinction of multiple geographically widespread taxa over a relatively short period of time^50^. There have been five major mass extinctions in the past 541 Myr^51,52^, with many arguing that human activity is currently causing a sixth^2^. In a similar way to our previous analysis of catastrophe rates, we should expect our upper bound to be consistent with the frequency of non-anthropogenic mass extinctions. Using only the big five extinctions produces a frequency of less than one per 100 Myr, far below our upper bound. In addition to the big five, there have been 13 other mass extinctions in the fossil record^53^. Using these numbers for 18 mass extinctions over 541 Myr still results in a frequency of about one per 30 Myr, many orders of magnitude below our upper bound.

## Conclusions

Using the fact that humans have survived at least 200 kyr, we can infer that the annual probability of human extinction from natural causes is less than 1 in 87,000 with modest confidence (0.1 relative likelihood) and less than 1 in 14,000 with near certainty (10^−6^ relative likelihood). These are the most conservative bounds. Estimates based on older fossils such as the ones found in Morocco dated to 315 kya result in annual extinction probabilities of less than 1 in 137,000 or 1 in 23,000 (for relative likelihood of 0.1 and 10^−6^, respectively). Using the track record of survival for the entire lineage of *Homo*, the annual probability of extinction from natural causes falls below 1 in 870,000 (relative likelihood of 0.1). We also conclude that these data are unlikely to be biased by observer selection effects, especially given that the bounds are consistent with mammalian extinction rates, the temporal range of other hominin species, and the frequency of potential catastrophes and mass extinctions.

The bounds are subject to important limitations. Most importantly, they only apply to extinction risks that have either remained constant or declined over human history. Our 200 kyr track record of survival cannot rule out much higher extinction probabilities from modern sources such as nuclear weapons or anthropogenic climate change. Some naturally occurring risks could be also be worsened by anthropogenic factors: a minor asteroid impact could be interpreted as a nuclear attack and lead to retaliation^54^, or a naturally occurring disease which previously may have only been a local extinction risk could spread much further due to modern travel^23^. In the cases where a natural risk is amplified by modern conditions, we can still derive some partial information from the upper bound by evaluating how much the risk would need to change from the purely natural baseline. For example, the claim that a natural disease poses a greater than 1 in 1,000 chance of extinction per year would require that anthropogenic conditions have increased the risk of natural disease by a factor of more than 14 to 870 (under our most conservative and optimistic upper bounds, respectively). In general, for a naturally occurring risk to violate our upper bounds via human activity by more than a factor of two, the majority of the risk would still need to come from anthropogenic circumstances.

In general, we conclude that anthropogenic extinction risks are likely greater than natural ones. We do not have a long track record of data for anthropogenic risks, so evaluating this relies far more on speculation. But despite the paucity of data, the little evidence we do have seems to be indicative of rates greatly exceeding our upper bounds. During the Cuban Missile Crisis of 1962, John F Kennedy put the odds of nuclear war at ‘somewhere between one out of three and even’^55^. If 0.1% of nuclear wars result in human extinction via nuclear winter, taking Kennedy’s odds that year would surpass our most conservative bound by more than a factor of four (and surpass our most optimistic bound by a factor of more than 250). Anthropogenic climate change could pose existential risks as well if warming is much worse than expected. A ballpark suggestion for the probability of 20 degrees of anthropogenic climate change was placed at 1%^56^, which would make the planet largely uninhabitable for humans due to heat stress^57^. And these are not the only risks we may face. One century ago, the existential risks posed by nuclear weapons or climate change may have seemed extremely implausible. We should therefore be cautious before dismissing the potential risks that future centuries of technological development could bring, such as those stemming from biotechnology^58^ or artificial general intelligence^59^.

Despite the low probability of human extinction from natural causes, it may still be prudent to reduce these risks. Existential risks jeopardize not only the lives of those currently present, but also the existence of all future generations. Depending on how much value we place on such generations, it may still be cost-effective to reduce existential risks from natural sources^60^. However, given limited resources to spend on reducing existential risks, one may be better off focusing on greater risks from our own design.
