# Author Correction: An upper bound for the background rate of human extinction

**DOI:** 10.1038/s41598-019-55816-1

**Published:** 2019-12-11

**Authors:** Andrew E. Snyder-Beattie, Toby Ord, Michael B. Bonsall

**Affiliations:** 1 University of Oxford, Mathematical Ecology Research Group, Department of Zoology, Oxford, OX1 3SZ UK; 2 University of Oxford, Future of Humanity Institute, Faculty of Philosophy, Oxford, OX1 1PT UK

Correction to: *Scientific Reports* 10.1038/s41598-019-47540-7, published online 30 July 2019

The Acknowledgements section in this Article is incomplete.

“We thank Carl Shulman, Dave Waltham, and Nick Bostrom for feedback and comments. This work was funded by Jaan Tallinn and the Open Philanthropy Project.”

should read:

“We thank Carl Shulman, Dave Waltham, and Nick Bostrom for feedback and comments. This work was funded by Jaan Tallinn and the Open Philanthropy Project. This project has received funding from the European Research Council (ERC) under the European Union’s Horizon 2020 research and innovation programme (grant agreement No 669751).”

